# Influence of head and neck position on the performance of supraglottic airway devices: A systematic review and meta-analysis

**DOI:** 10.1371/journal.pone.0216673

**Published:** 2019-05-09

**Authors:** Min-Soo Kim, Jin Ha Park, Ki-Young Lee, Seung Ho Choi, Hwan Ho Jung, Ji-Ho Kim, Bora Lee

**Affiliations:** 1 Department of Anesthesiology and Pain Medicine, Severance Hospital and Anesthesia and Pain Research Institute, Yonsei University College of Medicine, Seoul, Republic of Korea; 2 Department of Anesthesiology and Pain Medicine, National Health Insurance Service Ilsan Hospital, Goyang City, Gyeonggi-do, Republic of Korea; University of Oxford, UNITED KINGDOM

## Abstract

**Background:**

Changes in head and neck position may significantly affect the performance of supraglottic airway devices (SADs) by altering the pharyngeal structure.

**Purpose:**

This systematic review and meta-analysis aimed to elucidate the effect of changes in head and neck position on performance of SADs.

**Data source:**

Bibliographic databases, including PubMed, EMBASE, the Cochrane library, and the Web of Science.

**Study eligibility criteria:**

Prospective studies investigating the effects of head and neck positions on the performance of SADs.

**Methods:**

A random effect model was applied in the all analyses. Subgroup analysis was performed according to the type of device and the age of patient. The oropharyngeal leak pressure was the primary outcome measure. Secondary outcome measures included peak inspiratory pressure, fibreoptic view, and ventilation score (PROSPERO, CRD42017076971).

**Results:**

Seventeen studies met the eligibility criteria. Overall, the oropharyngeal leak pressure significantly increased (mean difference 4.07 cmH_2_O; 95% confidence interval 3.30 to 4.84) during neck flexion with adverse effects on ventilation and fibreoptic view. Conversely, the oropharyngeal leak pressure decreased (mean difference *−*4.05; 95% confidence interval *−*4.90 to *−*3.20) during neck extension with no significant effect on ventilation or fibreoptic view. Rotation of the head and neck did not significantly affect SAD performance.

**Conclusions:**

The reduced oropharyngeal leak pressure in the extended neck position was not associated with impaired ventilation except with the air-Q self-pressurizing airway. The flexed neck position significantly worsens ventilation and the alignment between the SAD and glottis despite improving the seal except with the air-Q self-pressurizing airway and LMA Proseal.

## Introduction

Supraglottic airway devices (SADs) are frequently used in lieu of tracheal intubation for patients undergoing elective procedures under general anesthesia.[1–5] Compared with tracheal intubation, the use of SADs has been shown to reduce the incidence of postoperative pharyngolaryngeal complications and shorten recovery time from anesthesia.[4] Despite these advantages, the performance of SADs can be affected by head and neck position.[5,6]

The volume and shape of the pharyngeal space changes significantly with changes in head and neck position.[4,5,7] The flexed neck position reduces the pharyngeal anteroposterior diameter by eliminating the longitudinal tension in the anterior pharyngeal muscles. Conversely, the extended neck position increases the anteroposterior diameter of the pharynx by elevating the laryngeal inlet.[4,8] Because the pharyngeal anatomy changes according to the head and neck position, the performance of SADs may be affected, including the sealing function, ventilation, and fibreoptic view.[4,9–12]

Since the laryngeal mask airway (LMA) Classic was first introduced, several other types of SADs have been developed and utilised in clinical practice.[1,4,9,13,14] Although the design of most SADs is generally based on the LMA Classic, each device has its own unique structural characteristics.[4,8,12,15] In addition, newer SADs, such as the i-gel and the air-Q self-pressurizing airway, do not require cuff inflation.[4,14]

Several studies have reported changes in SAD performance according to head and neck position but with conflicting results.[4,12,16,17] Therefore, this systematic review and meta-analysis aimed to integrate the existing data to elucidate the effect of head and neck position on the overall performance of SADs, as well as the performance of individual devices.

## Materials and methods

This systematic review and meta-analysis was reported in accordance with the Preferred Reporting Items for Systematic Reviews and Meta-Analyses (PRISMA) guidelines.[18] The protocol was registered with the International Prospective Registry of Systematic Reviews (PROSPERO, CRD42017076971) on September 14, 2017.

### Eligibility criteria

The studies included in the meta-analysis were prospective trials, including those with a crossover design, which investigated the influence of the various head and neck positions on the performance of SADs. The participants of studies included in this review were pediatric and adult patients who underwent general anesthesia with an SAD for airway management. The comparator was neutral head and neck position and intervention was changes of head and neck position, including flexion, extension, and rotation. The primary outcome measure was the oropharyngeal leak pressure. Secondary outcome measures were peak inspiratory pressure, fibreoptic view grade, and ventilation score. We included the studies which have the primary outcome.

### Literature search

We performed a computerised, systematic search of PubMed, EMBASE, the Cochrane library, and the Web of Science on October 17, 2017, using combinations of the following search terms: head, neck, position, positions, extension, rotation, flexion, supraglottic airway, laryngeal mask, i-gel, air-Q, and laryngeal tube. The search strategy created for use in PubMed was ((((((((((head) OR neck)) AND ((positions) OR position))) OR extension) OR rotation) OR flexion)) AND (((((supraglottic airway) OR laryngeal mask) OR i-gel) OR air-Q) OR laryngeal tube)). No age or language restrictions were applied to article selection. Published data that did not belong to general journal articles, such as letters, editorials, and conference papers, were excluded. Search results derived from each database were integrated and duplicates were eliminated. Two authors (K.-Y.L., S.H.C.) independently performed a primary screening of the search results based on article titles and abstracts. The same authors then conducted a full-text assessment of articles for final inclusion. Disagreements regarding study inclusion were resolved by discussion and consensus, and uncertainties were resolved by consulting a third author (J.-H. K.). In addition, the reference lists of all included articles were investigated to find other potentially eligible studies.

### Risk of bias assessment

Two authors (B.L. and H.H.J.) independently assessed the quality of the included studies using the Cochrane Collaboration Risk of Bias Tool, which evaluates several domains of bias, including selection, performance, detection, attrition, reporting, and other forms of bias.[19] Each bias domain was graded as high risk, low risk, or unclear. Disagreements regarding bias were resolved through discussion and consensus, and uncertainties were resolved by consulting a third author (J.H.P.).

### Data collection

Two authors (J.H.P. and M.-S.K.) independently collected relevant data from the final selected studies. Disagreements regarding data collection were resolved by discussion and consensus, and uncertainties were resolved by consulting a third author (B.L.). The authors extracted the following data: the primary author’s name, publication year, study design, sample size, patient characteristics, type of SAD, details of interventions, primary and secondary outcome measures, conflict of interests, and funding source. In addition, we emailed the study authors to request for raw data in order to compute missing values and correlation coefficients. The ventilation quality scores were reversed for comparisons with the ventilation scores.

When the data were described as median, total range, and interquartile range (IQR), the mean value and standard deviations were estimated using formulae developed by Wan and colleagues.[20] If the value was provided as 95% confidence intervals, the standard deviations were calculated inversely using the confidence interval formula with *t*-distribution.

### Statistical analysis

Data were analysed using R version 3.4.1 (R Foundation for Statistical Computing, Vienna, Austria). We calculated the individual values and pooled estimates of the raw mean difference with 95% confidence interval for continuous outcomes and the risk ratio (RR) with 95% confidence interval for dichotomous outcomes. Correlation coefficients used to compute estimates for each study were computed from the raw data provided by the study authors. If raw data were unavailable, correlation coefficients obtained from the studies with available raw data were applied. The I^2^ statistic was used to assess heterogeneity using the following predetermined thresholds: low heterogeneity (I^2^ = 25–49%), moderate heterogeneity (I^2^ = 50–74%), and high heterogeneity (I^2^ > 75%).[21] A random effect model with the DerSimonian and Laird method was applied in the all analyses. Subgroup analysis was performed according to the type of device and the age of patient. Publication bias was assessed by visually examining the asymmetry of a funnel plot and using Egger’s linear regression test.[22] The presence of publication bias was suspected when the P-value of the Egger test was less than 0.1.

## Results

**Fig 1:**
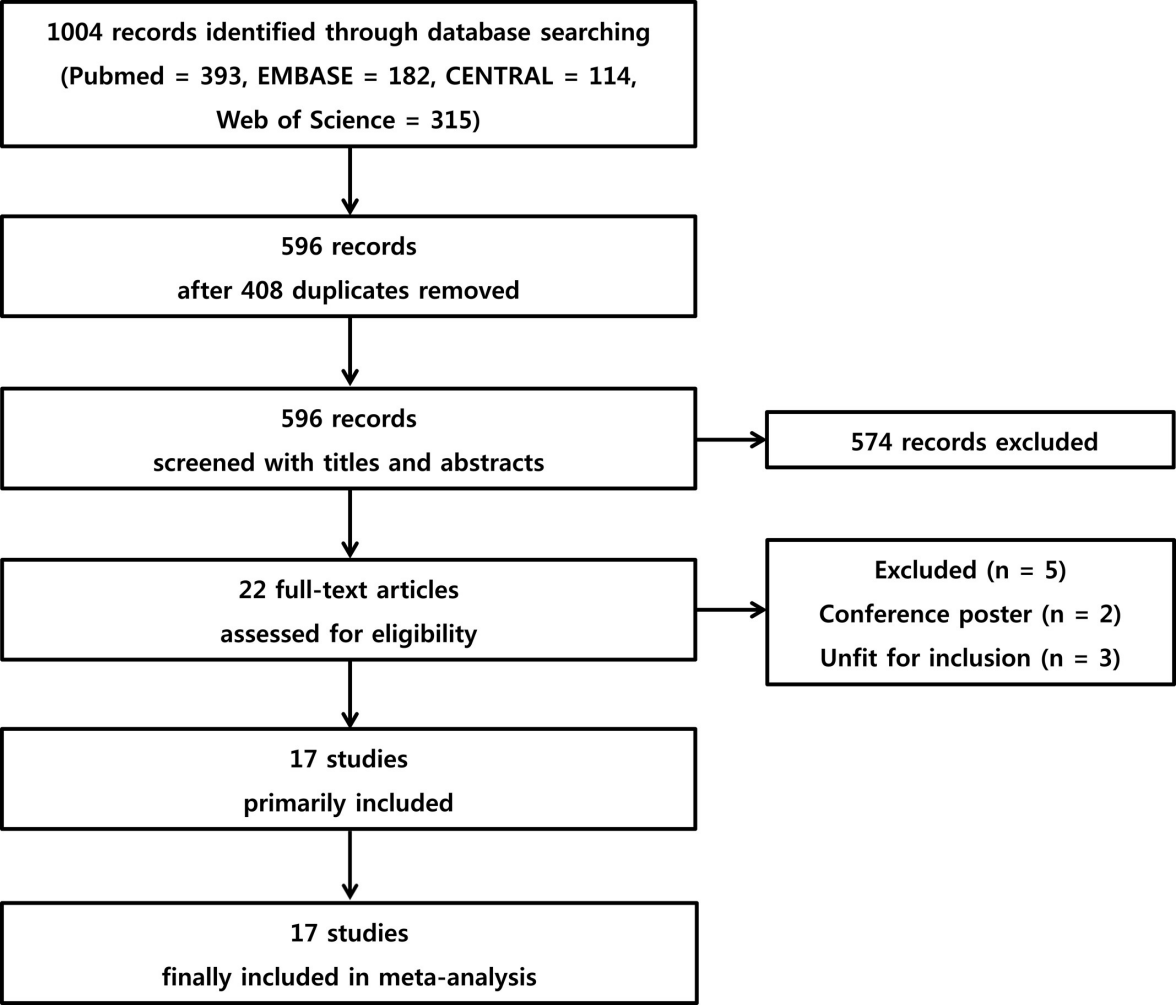
Flow chart of the literature screening process.

Of the 1004 articles retrieved from the literature search, 17 studies met the eligibility criteria, representing a total of 959 patients (Fig 1). A full description of the characteristics of the included studies is provided in Table 1.

**Table 1:** Characteristics of the included trials.

	Devices	N	Age [yr; mean _(SD)_]	Interventions	Outcome collected	COI
Kim, 2017	air-Q self-pressurizing airway	51	43 (10)	Flexion, extension, rotation with NMB	oropharyngeal leak pressure, peak inspiratory pressure, VS, fibreoptic view	No
Gupta, 2017	i-gel	30	3.91, Mean	Flexion, extension without NMB	oropharyngeal leak pressure, fibreoptic view	No
	Supreme	30				
Somri, 2016	Supreme	80	55.1 (11.3)	Flexion, extension, right/left rotation with NMB	oropharyngeal leak pressure	No
	Laryngeal tube suction	80	55.5 (13.3)			
Jain, 2016	i-gel	60	5.1 (2.1)	Flexion 15°, 30°, 45° with NMB	oropharyngeal leak pressure, peak inspiratory pressure, VS, fibreoptic view	No
Mishra, 2015	Proseal	30	38 (14.3)	Flexion, extension, rotation with NMB	oropharyngeal leak pressure, peak inspiratory pressure, VS, fibreoptic view	No
	i-gel	30	38 (13.1)			
Jain, 2015	i-gel	30	4.5 [3.8–5.6] median [95% confidence interval]	Flexion, extension with NMB	oropharyngeal leak pressure, peak inspiratory pressure, VS, fibreoptic view	No
Biedler, 2013	LT	17	3.8 (2.1)	Flexion, extension, right/left rotation without NMB	oropharyngeal leak pressure, peak inspiratory pressure	-
	Classic	22	3.5 (2.1)			
Mann, 2012	Proseal	27/26	32 [23; 44]	Flexion, extension, right/left rotation with/without NMB	oropharyngeal leak pressure	No
	Laryngeal tube suction	26/26	37 [26; 46] median [IQR]			
Sanuki, 2011	i-gel	20	32.4 (15.0)	Flexion, extension, rotation with NMB	oropharyngeal leak pressure, peak inspiratory pressure, VS, fibreoptic view	No
Park, 2009	Proseal	45/45	43 (13)	Flexion, extension, rotation with NMB	oropharyngeal leak pressure, fibreoptic view	-
	Laryngeal tube suction	38/45	42 (14)			
	Corbra perilaryngeal airway	45/45	46 (16)			
Kim, 2009	Laryngeal tube suction	33	7.1 (3.0)	Flexion, extension, rotation with NMB	oropharyngeal leak pressure, peak inspiratory pressure, VS, fibreoptic view	No
Xue, 2008	Proseal	80	18–57 range	Flexion with NMB	oropharyngeal leak pressure, VS, fibreoptic view	-
Choi, 2007	Proseal	29	4.3 (2.0)	Rotation with NMB	oropharyngeal leak pressure, fibreoptic view	-
Brimacombe, 2003	Proseal	30	37 (18–67), mean (range)	Flexion, extension, rotation with NMB	oropharyngeal leak pressure, fibreoptic view	-
	Classic					
Okuda, 2001	Classic	39	4.0 (1.5–8.0), mean (range)	Flexion, extension, right/left rotation without NMB	oropharyngeal leak pressure, fibreoptic view	-
Keller, 1999	Classic	20	36 [30–42], mean [95% confidence interval]	Flexion, extension, rotation with NMB	oropharyngeal leak pressure, fibreoptic view	-
	Flexible					
Buckham, 1999	Classic	60	38 (13)	Flexion, extension, rotation with NMB	oropharyngeal leak pressure	-
	Flexible					

N, number; SD, standard deviation; COI, conflict of interests; NMB, neuromuscular blocking agent; oropharyngeal leak pressure, oropharyngeal leak pressure; peak inspiratory pressure, peak inspiratory pressure; VS, ventilation score; Supreme, laryngeal mask airway Supreme; laryngeal tube suction, laryngeal tube suction; Proseal, laryngeal mask airway Proseal; LT, laryngeal tube; confidence interval, confidence interval; LT, laryngeal tube; Classic, laryngeal mask airway Classic; IQR, interquartile range; corbra perilaryngeal airway, Cobra Perilaryngeal Airway; Flexible, laryngeal mask airway Flexible.

### Study characteristics

SADs used for the included studies were as follows: the air-Q self-pressurizing airway was used in 1 study,[4] the i-gel was used in 5 studies,[10–12,14,23] the LMA Supreme was used in 2 studies,[6,23] the laryngeal tube was used in 1 study,[9] the laryngeal tube suction was used in 4 studies,[6,8,17,24] the LMA Proseal was used in 6 studies,[8,11,15,16,24,25] the Cobra perilaryngeal airway was used in 1 study,[8] the LMA Classic was used in 5 studies,[5,7,9,13,25] and the LMA Flexible was used in 2 studies.[7,13] The reinforced LMA was regarded as the LMA Flexible.[13] Seven studies investigated SADs in pediatric patients,[5,9,10,12,15,17,23] and 4 studies did not use neuromuscular blocking agents.[5,9,23,24] Oropharyngeal leak pressure was defined as the airway pressure at which a leak sound was detected around the patient’s mouth and at which the airway pressure had reached equilibrium, when the pressure-limiting valve of the anesthesia breathing system was closed and the fresh gas flow rate was fixed at 3 L/min.[2] Peak inspiratory pressure was measured using a respirator and was indicated on the display during mechanical ventilation.[9] Seven studies recorded data of the peak inspiratory pressure at a tidal volume of 10 mL/kg [4,10–12,14,17] or 8 mL/kg.[9] Thirteen studies reported data on the fibreoptic view, wherein the Brimacombe score[26] was used in 8 studies,[4,7,8,11,14–16,25] the Okuda score[5] was used in 3 studies,[5,10,12] the Cook score[1] was used in 1 study,[17] and the 5-point score was used in 1 study.[23] The best and worst fibreoptic view ratings of the devices in each study was used for this meta-analysis. One study abstained from evaluating the fiberoptic score if patients experienced ventilatory difficulty after neck motion.[8] Seven studies provided data regarding the ventilation score[4,10–12,14,17] or quality.[16] The ventilation score was rated from 0 to 3 based on three criteria (no leakage at an airway pressure of 15 cmH_2_O, bilateral chest excursion with 20 cmH_2_O of peak inspiratory pressure, and a square-wave capnogram).[3] The ventilation quality was assessed with a 3-point ventilation score, wherein 1 = chest expansion without gas leakage; 2 = chest expansion with obvious gas leakage; and 3 = minimal chest expansion and considerable gas leakage.[2,16] In studies in which data were reported in both the left- and right-rotated neck positions, we selected data measured in the right-rotated position.[5,6,9,24] One study was published in German with an abstract available in English.[24] After translating the article to English, we performed data collection and bias evaluation from this included study. Raw data provided by Kim and colleagues was used to determine correlation coefficients and compute effect sizes for the oropharyngeal leak pressure and peak inspiratory pressure.[4] Effect sizes for the fibreoptic view and ventilation score were estimated as two independent groups, without applying correlation coefficients. All of the included studies were graded using the Cochrane Collaboration Risk of Bias Tool (Table 2).

**Table 2:** Assessment of bias risk items for each included study.

	Random sequence generation	Allocation concealment	Blinding of participants and personnel	Blinding of outcome assessment	Incomplete outcome data	Selective reporting	Other bias
Kim, 2017	low	low	unclear	high	low	low	unclear
Gupta, 2017	unclear	high	unclear	unclear	low	low	low
Somri, 2016	low	low	unclear	unclear	low	low	low
Jain, 2016	unclear	high	unclear	high	high	low	unclear
Mishra, 2015	low	low	unclear	unclear	unclear	unclear	unclear
Jain, 2015	low	low	unclear	unclear	low	low	unclear
Biedler, 2013	low	unclear	unclear	unclear	low	low	unclear
Mann, 2012	low	low	unclear	unclear	low	low	low
Sanuki, 2011	unclear	unclear	unclear	unclear	low	low	low
Park, 2009	low	low	low	low	high	low	low
Kim, 2009	unclear	unclear	low	low	low	low	unclear
Xue, 2008	low	low	low	low	low	low	low
Choi, 2007	high	high	unclear	unclear	low	low	unclear
Brimacombe, 2003	low	low	low	low	low	low	unclear
Okuda, 2001	unclear	high	low	low	low	low	unclear
Keller, 1999	unclear	unclear	unclear	low	low	low	low
Buckham, 1999	low	low	unclear	unclear	low	low	low

Low, low risk of bias; High, high risk of bias; Unclear, unclear risk of bias.

### Oropharyngeal leak pressure (Table 3)

**Table 3:** Meta-analysis of oropharyngeal leak pressure.

Group or subgroup	Number of comparisons	MD (95% CI)	I^2^	*P*	*P* in Egger’s Test
*Flexion—Neutral*					
Overall analysis	28	4.07 (3.30 to 4.84)	92.8%	< 0.001	0.403
air-Q SP	1	4.00 (3.23 to 4.77)	-	< 0.001	-
Classic	5	5.98 (4.98 to 6.97)	34.1%	< 0.001	0.043
CobraPLA	1	2.40 (1.30 to 3.49)	-	< 0.001	-
Flexible	2	6.09 (2.17 to 10.00)	92.4%	0.002	-
i-gel	5	4.17 (3.08 to 5.25)	90.0%	< 0.001	0.068
LT	1	3.80 (1.34 to 6.26)	-	0.003	-
LTS	5	0.75 (-0.84 to 2.33)	80.6%	0.357	0.802
Proseal	6	5.36 (3.97 to 6.75)	82.0%	< 0.001	0.395
Supreme	2	2.97 (1.15 to 4.79)	90.7%	0.001	-
Adult patients	20	4.03 (2.94 to 5.13)	93.6%	< 0.001	0.429
Paediatric patients	8	4.14 (3.07 to 5.20)	90.2%	< 0.001	0.640
*Extension-Neutral*					
Overall analysis	26	-4.05 (-4.90 to -3.20)	83.0%	< 0.001	0.800
air-Q SP	1	-8.60 (-10.07 to -7.13)	-	< 0.001	-
Classic	5	-3.91 (-6.41 to -1.40)	80.2%	0.002	0.249
CobraPLA	1	-1.90 (-3.57 to -0.23)	-	0.026	-
Flexible	2	-4.41 (-6.20 to -2.63)	42.9%	< 0.001	-
i-gel	4	-3.40 (-3.99 to -2.82)	0.0%	< 0.001	0.512
LT	1	-1.80 (-5.71 to 2.11)	-	0.367	-
LTS	5	-3.57 (-6.11 to -1.04)	78.5%	0.006	0.645
Proseal	5	-6.31 (-8.42 to -4.20)	60.7%	< 0.001	0.513
Supreme	2	-2.16 (-2.76 to -1.57)	0.0%	< 0.001	-
Adult patients	19	-4.64 (-5.78 to -3.51)	83.4%	< 0.001	0.786
Paediatric patients	7	-2.80 (-3.55 to -2.04)	41.6%	< 0.001	0.420
*Rotation-Neutral*					
Overall analysis	24	0.55 (-0.05 to 1.15)	82.2%	0.072	0.348
air-Q SP	1	-0.20 (-0.80 to 0.40)	-	0.516	-
Classic	5	2.17 (1.29 to 3.06)	41.4%	<0.001	0.725
CobraPLA	1	-0.20 (-1.29 to 0.89)	-	0.719	-
Flexible	2	-0.96 (-4.87 to 2.96)	96.1%	0.633	-
i-gel	2	0.17 (-0.54 to 0.89)	0.0%	0.636	-
LT	1	1.00 (-1.26 to 3.26)	-	0.387	-
LTS	5	-0.34 (-1.60 to 0.91)	64.8%	0.595	0.815
Proseal	6	1.01 (-0.42 to 2.43)	84.0%	0.166	0.307
Supreme	1	-1.00 (-1.90 to -0.10)	-	0.030	-
Adult patients	19	0.26 (-0.41 to 0.94)	83.2%	0.441	0.489
Paediatric patients	5	1.56 (0.96 to 2.16)	0.00%	< 0.001	0.515

The MD was measured in cmH_2_O according to the position listed compared to neutral. MD, mean difference; CI, confidence interval; air-Q SP, air-Q self-pressurizing airway; Classic, laryngeal mask airway Classic; CobraPLA, Cobra Perilaryngeal Airway; Flexible, laryngeal mask airway Flexible; LT, laryngeal tube; LTS, laryngeal tube suction; Proseal, laryngeal mask airway Proseal; Supreme, laryngeal mask airway Supreme.

**Fig 2:**
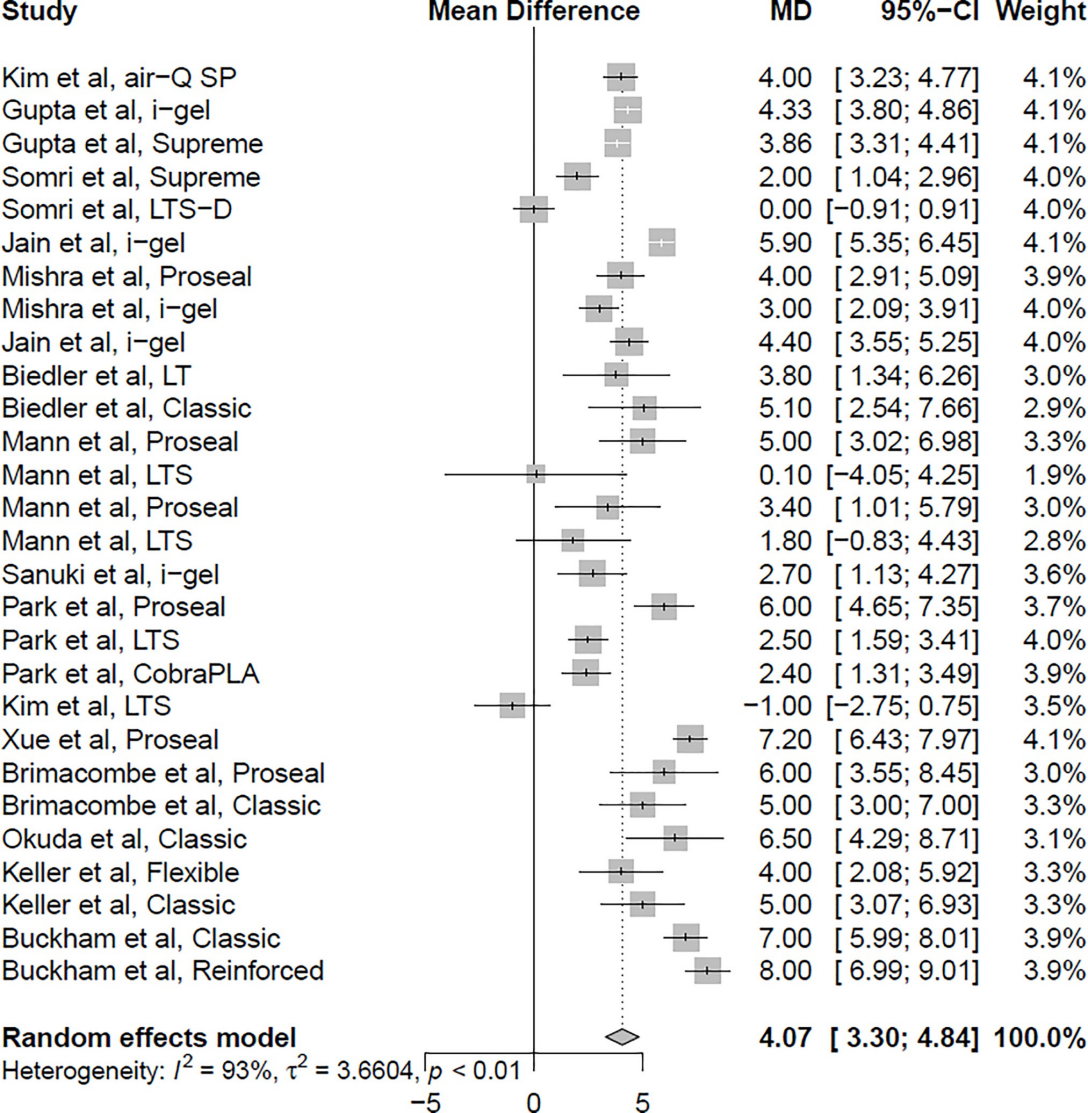
Forest plot of the oropharyngeal leak pressure in the flexed neck position compared with the neutral neck position.

**Fig 3:**
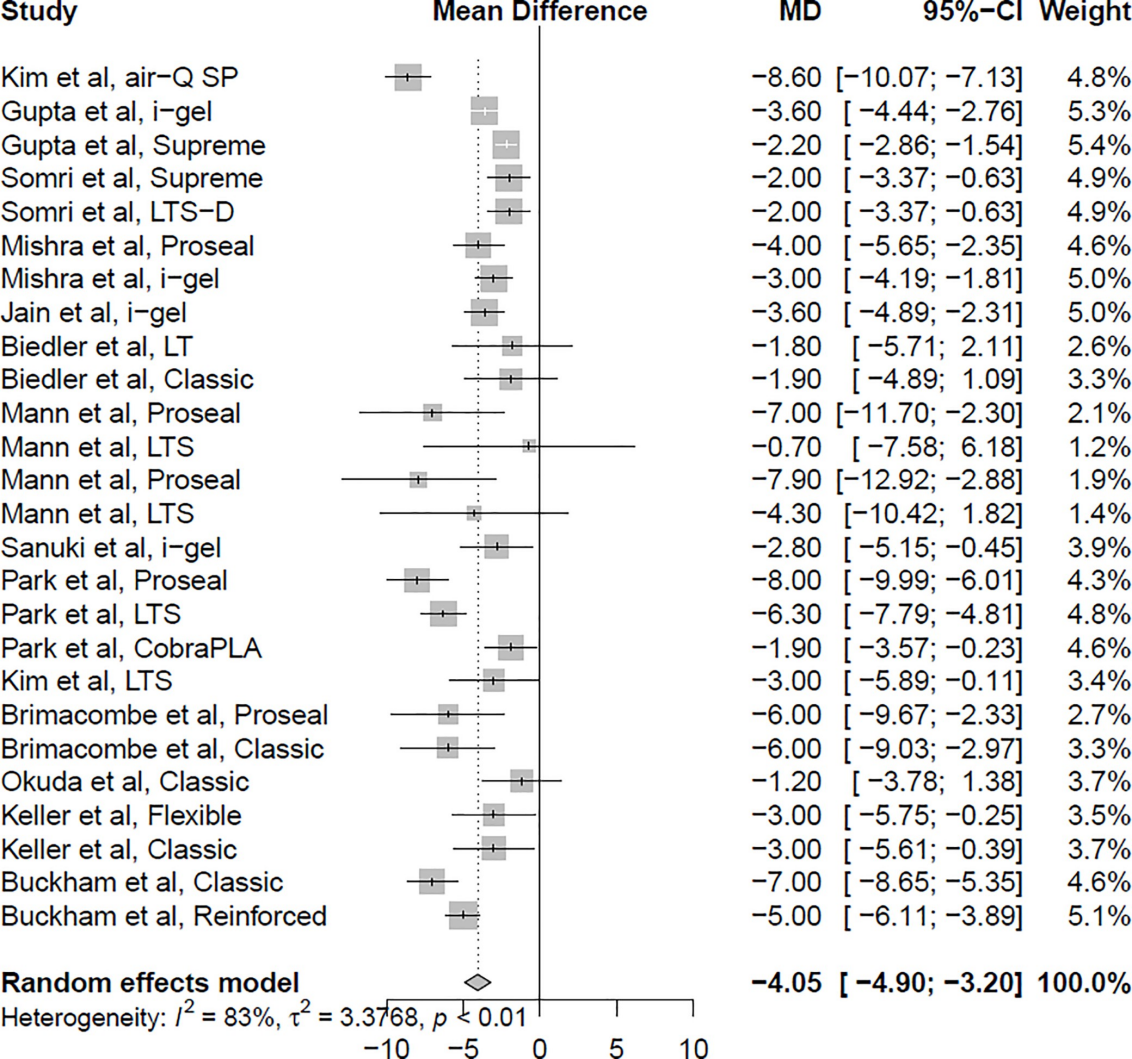
Forest plot of the oropharyngeal leak pressure in the extended neck position compared with the neutral neck position.

Sixteen studies reported data comparing the oropharyngeal leak pressure between the flexed and neutral neck positions.[4–14,16,17,23–25] The overall analysis showed a significantly higher oropharyngeal leak pressure in the flexed neck position than in the neutral neck position (mean difference 4.07 cmH_2_O; 95% confidence interval 3.30 to 4.84; I^2^ = 92.8%; *P* < 0.001) (Fig 2). Fourteen studies reported data comparing the oropharyngeal leak pressure between the extended and neutral neck positions.[4–11,13,14,17,23–25] The overall analysis revealed that the oropharyngeal leak pressure was significantly lower in the extended neck position than in the neutral neck position (mean difference *−*4.05; 95% confidence interval *−*4.90 to *−*3.20; I^2^ = 83.0%; *P* < 0.001) (Fig 3). A greater reduction in the oropharyngeal leak pressure was observed during neck extension with the air-Q self-pressurizing airway (mean difference *−*8.60; 95% confidence interval *−*10.07 to *−*7.13),[4] the LMA Proseal (mean difference *−*6.31; 95% confidence interval *−*8.42 to *−*4.20),[8,11,24,25] and the LMA Flexible (mean difference *−*4.41; confidence interval *−*6.20 to *−*2.63).[7,13] Subgroup analysis by patient age revealed that the decrease in oropharyngeal leak pressure during neck extension was greater in adult patients (mean difference *−*4.64; 95% confidence interval *−*5.78 to *−*3.51)[4,6–8,11,13,14,24,25] than in pediatric patients (mean difference *−*2.80; 95% confidence interval *−*3.55 to *−*2.04).[5,9,10,17,23]

Thirteen studies reported data comparing the oropharyngeal leak pressure between the rotated and neutral neck positions.[4–9,11,13–15,17,24,25] The overall analysis showed no significant difference in the oropharyngeal leak pressure between the rotated and the neutral neck positions. In subgroup analyses, the oropharyngeal leak pressure was significantly higher in the rotated neck position with the LMA Classic (mean difference 2.17; 95% confidence interval 1.29 to 3.06; I^2^ = 41.4%; *P* < 0.001),[5,7,9,13,25] as well as in the pediatric subgroup (mean difference 1.56; 95% confidence interval 0.96 to 2.16; I^2^ = 0.0%; *P* < 0.001).[5,9,15,17]

### Peak inspiratory pressure (Table 4)

**Table 4:** Meta-analysis of peak inspiratory pressure (cmH_2_O).

Group or subgroup	Number of comparisons	MD (95% CI)	I^2^	*P*	*P* in Egger’s Test
*Flexion—Neutral*					
Overall analysis	9	5.18 (3.81 to 6.55)	95.6%	< 0.001	0.807
air-Q SP	1	3.10 (2.10 to 4.10)	-	< 0.001	-
Classic	1	3.60 (1.72 to 5.48)	-	< 0.001	-
i-gel	4	4.54 (3.56 to 5.53)	90.0%	< 0.001	0.032
LT	1	6.60 (4.31 to 8.89)	-	< 0.001	-
LTS	1	13.00 (11.62 to 14.38)	-	< 0.001	-
Proseal	1	3.00 (1.37 to 4.63)	-	< 0.001	-
Adult patients	4	3.34 (1.99 to 4.70)	80.0%	< 0.001	0.498
Paediatric patients	5	6.71 (4.80 to 8.62)	96.9%	< 0.001	0.589
*Extension-Neutral*					
Overall analysis	8	-2.23 (-3.21 to -1.26)	89.8%	< 0.001	0.002
air-Q SP	1	-1.80 (-2.31 to -1.29)	-	< 0.001	-
Classic	1	-1.50 (-2.84 to -0.16)	-	0.028	-
i-gel	3	-1.58 (-3.42 to 0.26)	93.3%	0.093	0.435
LT	1	-6.80 (-9.14 to -4.46)	-	< 0.001	-
LTS	1	-4.00 (-5.17 to -2.83)	-	< 0.001	-
Proseal	1	-1.00 (-1.71 to -0.29)	-	0.006	-
Adult patients	4	-1.58 (-2.79 to -0.37)	90.7%	0.011	0.603
Paediatric patients	4	-3.17 (-5.19 to -1.14)	91.0%	0.002	0.021
*Neutral-rotation*					
Overall analysis	7	-0.79 (-1.76 to 0.17)	96.1%	0.108	0.005
air-Q SP	1	-0.20 (-0.45 to 0.05)	-	0.115	-
Classic	1	0.20 (-0.59 to 0.99)	-	0.618	-
i-gel	2	0.97 (0.60 to 1.35)	0.0%	< 0.001	-
LT	1	-4.20 (-5.45 to -2.95)	-	< 0.001	-
LTS	1	-4.00 (-4.85 to -3.15)	-	< 0.001	-
Proseal	1	0.00 (-0.35 to 0.35)	-	1.000	-
Adult patients	4	0.38 (-0.21 to 0.97)	88.9%	0.208	0.050
Paediatric patients	3	-2.65 (-5.68 to 0.39)	96.8%	0.087	0.412

MD, mean difference; CI, confidence interval; air-Q SP, air-Q self-pressurizing airway; Classic, laryngeal mask airway Classic; LT, laryngeal tube; LTS, laryngeal tube suction; Proseal, laryngeal mask airway Proseal.

Seven studies reported data comparing the peak inspiratory pressure between the flexed and neutral neck positions.[4,9–12,14,17] The overall analysis revealed a significantly higher peak inspiratory pressure in the flexed neck position than in the neutral neck position (mean difference 5.18; 95% confidence interval 3.81 to 6.55; I^2^ = 95.6%; *P* < 0.001). The increase in peak inspiratory pressure was greater in pediatric patients (mean difference 6.71; 95% confidence interval 4.80 to 8.62)[9,10,12,17] than in adult patients (mean difference 3.34; 95% 1.99 to 4.70).[4,11,14] Six studies compared the peak inspiratory pressure between the extended and neutral neck positions.[4,9–12,17] The overall analysis revealed a significantly lower peak inspiratory pressure in the extended neck position than in the neutral neck position (mean difference *−*2.23; 95% confidence interval *−*3.21 to *−*1.26; I^2^ = 89.6%; *P* < 0.001). Five studies reported data comparing the peak inspiratory pressure between the rotated and neutral neck positions.[4,9,11,14,17] Neither the overall or subgroup analyses showed any significant change in peak inspiratory pressure during neck rotation.

### Fibreoptic view evaluation (Tables 5 and 6)

**Table 5:** Meta-analysis of the worst fibreoptic view.

Group or subgroup	Number of comparisons	Risk Ratio (95% CI)	I^2^	*P*	*P* in Egger’s Test
*Flexion—Neutral*					
Overall analysis	15	1.70 (1.06 to 2.73)	40.8%	0.028	0.112
Classic	3	1.46 (0.51 to 4.22)	0.0%	0.485	0.113
CobraPLA	1	1.00 (0.44 to 2.29)	-	1.000	-
Flexible	1	1.50 (0.28 to 8.04)	-	0.636	-
i-gel	3	7.67 (1.75 to 33.70)	0.0%	0.007	0.145
LTS	2	2.91 (0.94 to 9.01)	59.1%	0.063	-
Proseal	4	0.96 (0.62 to 1.48)	0.0%	0.856	0.185
Supreme	1	1.00 (0.07 to 15.26)	-	1.000	-
Adult patients	10	1.04 (0.74 to 1.46)	0.0%	0.8351	0.072
Paediatric patients	5	4.59 (2.36 to 8.95)	0.0%	< 0.001	0.840
*Extension-Neutral*					
Overall analysis	12	0.71 (0.43 to 1.17)	0.0%	0.180	0.816
Classic	3	0.84 (0.26 to 2.69)	0.0%	0.769	0.024
CobraPLA	1	0.67 (0.26 to 1.72)	-	0.401	-
Flexible	1	1.00 (0.16 to 6.42)	-	1.000	-
i-gel	1	2.0 (0.19 to 20.90)	-	0.563	-
LTS	2	0.44 (0.14 to 1.38)	0.0%	0.162	-
Proseal	3	0.81 (0.27 to 2.43)	0.0%	0.701	0.227
Supreme	1	0.33 (0.01 to 7.86)	-	0.496	-
Adult patients	9	0.76 (0.44 to 1.32)	0.0%	0.333	0.968
Paediatric patients	3	0.54 (0.17 to 1.67)	0.0%	0.284	0.935
*Rotation-Neutral*					
Overall analysis	11	0.81 (0.50 to 1.33)	0.0%	0.412	0.355
Classic	3	0.84 (0.26 to 2.69)	0.0%	0.769	0.024
CobraPLA	1	0.89 (0.38 to 2.10)	-	0.788	-
Flexible	1	1.00 (0.16 to 6.42)	-	1.000	-
i-gel	1	2.00 (0.19 to 20.90)	-	0.563	-
LTS	2	0.48 (0.14 to 1.66)	6.3%	0.249	-
Proseal	3	0.81 (0.27 to 2.43)	0.0%	0.701	0.227
Adult patients	9	0.88 (0.52 to 1.48)	0.0%	0.620	0.553
Paediatric patients	2	0.47 (0.10 to 2.32)	21.0%	0.357	-

Risk ratio indicates the ratio of probability of obtaining the worst score. CI, confidence interval; Classic, laryngeal mask airway Classic; CobraPLA, Cobra Perilaryngeal Airway; Flexible, laryngeal mask airway Flexible; LTS, laryngeal tube suction; Proseal, laryngeal mask airway Proseal; Supreme, laryngeal mask airway Supreme.

**Table 6:** Meta-analysis of the best fibreoptic view.

Group or subgroup	Number of comparisons	Risk Ratio (95% CI)	I^2^	*P*	*P* in Egger’s Test
*Flexion—Neutral*					
Overall analysis	17	0.76 (0.61 to 0.96)	38.9%	0.020	0.002
air-Q SP	1	0.80 (0.23 to 2.81)	-	0.728	-
Classic	3	0.87 (0.51 to 1.48)	32.3%	0.607	0.654
CobraPLA	1	0.90 (0.40 to 2.00)	-	0.796	-
Flexible	1	0.60 (0.17 to 2.18)	-	0.438	-
i-gel	5	0.46 (0.20 to 1.10)	85.5%	0.082	0.013
LTS	2	0.25 (0.01 to 5.58)	79.3%	0.382	-
Proseal	3	0.94 (0.64 to 1.37)	0.0%	0.749	0.498
Supreme	1	0.73 (0.41 to 1.32)	-	0.304	-
Adult patients	11	0.92 (0.78 to 1.08)	0.0%	0.289	0.245
Paediatric patients	6	0.37 (0.18 to 0.75)	59.4%	0.006	0.007
*Extension-Neutral*					
Overall analysis	16	1.08 (0.95 to 1.23)	0.0%	0.256	0.337
air-Q SP	1	1.00 (0.31 to 3.25)	-	1.000	-
Classic	3	1.06 (0.76 to 1.49)	0.0%	0.723	0.261
CobraPLA	1	1.20 (0.58 to 2.49)	-	0.625	-
Flexible	1	0.60 (0.17 to 2.18)	-	0.438	-
i-gel	4	0.98 (0.81 to 1.19)	0.0%	0.843	0.386
LTS	2	1.75 (0.86 to 3.55)	68.3%	0.121	-
Proseal	3	1.06 (0.74 to 1.53)	0.0%	0.733	0.228
Supreme	1	1.07 (0.65 to 1.74)	-	0.796	-
Adult patients	11	1.00 (0.85 to 1.16)	0.0%	0.957	0.612
Paediatric patients	5	1.38 (0.96 to 1.98)	46.7%	0.080	0.513
*Rotation-Neutral*					
Overall analysis	14	1.09 (0.88 to 1.34)	38.6%	0.428	0.958
air-Q SP	1	1.00 (0.31 to 3.25)	-	1.000	-
Classic	3	1.34 (0.79 to 2.27)	51.9%	0.276	0.722
CobraPLA	1	1.00 (0.46 to 2.17)	-	1.000	-
Flexible	1	0.60 (0.17 to 2.18)	-	0.438	-
i-gel	2	0.83 (0.42 to 1.64)	71.4%	0.596	-
LTS	2	1.66 (0.63 to 4.40)	82.3%	0.307	-
Proseal	4	0.91 (0.66 to 1.27)	0.0%	0.585	0.469
Adult patients	11	0.97 (0.83 to 1.12)	0.0%	0.662	0.267
Paediatric patients	3	1.71 (0.87 to 3.37)	73.6%	0.120	0.245

Risk ratio indicates the ratio of probability of obtaining the best score. CI, confidence interval; air-Q SP, air-Q self-pressurizing airway; Classic, laryngeal mask airway Classic; CobraPLA, Cobra Perilaryngeal Airway; Flexible, laryngeal mask airway Flexible; LTS, laryngeal tube suction; Proseal, laryngeal mask airway Proseal; Supreme, laryngeal mask airway Supreme.

Twelve studies reported data comparing the fibreoptic view between the flexed and neutral neck positions.[4,5,7,8,10–12,14,16,17,23,25] The overall analysis revealed that the flexed neck position significantly increased the incidence of receiving the worst fibreoptic view rating (RR 1.70; 95% confidence interval 1.06 to 2.73; I^2^ = 40.8%; *P* < 0.028)[5,7,8,10–12,16,17,23,25] and decreased the incidence of receiving the best fibreoptic view rating (RR 0.76; 95% confidence interval 0.61 to 0.96; I^2^ = 38.9%; *P* = 0.020).[4,5,7,8,10–12,14,17,23,25] Compared with other devices, the incidence of the worst fibreoptic view rating was significantly higher with the i-gel (RR 7.67; 95% confidence interval 1.75 to 33.70; I^2^ = 0.0%; *P* = 0.007).[10–12] In pediatric patients, the flexed neck position resulted in a substantially greater chance of receiving the worst fibreoptic view rating (RR 4.59; 95% confidence interval 2.36 to 8.95; I^2^ = 0.0%; *P* < 0.001)[5,10,12,17,23] and a significantly lower chance of receiving the best fibreoptic view rating (RR 0.37; 95% confidence interval 0.18 to 0.75; I^2^ = 59.4%; *P* = 0.006).[5,10,12,17,23]

Ten studies reported data comparing the fibreoptic view between the extended and neutral neck positions.[4,5,7,8,10,11,14,17,23,25] Neck extension did not significantly change the incidence of receiving the worst fibreoptic view rating in the overall or subgroup analyses.

Nine studies reported data comparing the fibreoptic view between the rotated and neutral neck positions.[4,5,7,8,11,14,15,17,25] The analysis of these studies revealed that the rotated neck position did not significantly affect the incidence of receiving either the worst or best fibreoptic view rating.

### Ventilation score (Table 7)

**Table 7:** Meta-analysis of ventilation score.

Group or subgroup	Number of comparisons	MD (95% CI)	I^2^	*P*	*P* in Egger’s Test
*Flexion—Neutral*					
Overall analysis	7	-0.74 (-1.20 to -0.28)	98.8%	0.002	0.002
Air-Q SP	1	-0.04 (-0.09 to 0.015)	-	0.153	-
i-gel	3	-1.25 (-1.72 to -0.79)	91.5%	< 0.001	0.671
LTS	1	-1.50 (-1.76 to -1.24)	-	< 0.001	-
Proseal	2	0.04 (-0.20 to 0.28)	81.3%	0.735	-
Adult patient	4	-0.14 (-0.36 to 0.07)	93.7%	0.191	0.012
Paediatric patient	3	-1.50 (-1.63 to -1.37)	0.0%	< 0.001	1.000
*Extension-Neutral*					
Overall analysis	5	-0.11 (-0.30 to 0.07)	92.3%	0.221	0.721
Air-Q SP	1	-0.55 (-0.69 to -0.41)	-	< 0.001	-
i-gel	3	-0.02 (-0.08 to 0.03)	0.0%	0.442	0.638
LTS	1	0.00 (-0.12 to 0.12)	-	1.000	-
Adult patient	3	-0.19 (-0.53 to 0.14)	95.8%	0.259	0.843
Paediatric patient	2	0.00 (-0.09 to 0.09)	0.0%	1.000	-
*Rotation-Neutral*					
Overall analysis	3	-0.02 (-0.05 to 0.02)	0.0%	0.344	0.713
Air-Q SP	1	-0.02 (-0.06 to 0.02)	-	0.308	-
i-gel	1	0.00 (-0.17 to 0.17)	-	1.000	-
LTS	1	0.00 (-0.12 to 0.12)	-	1.000	-
Adult patient	2	-0.02 (-0.06 to 0.02)	0.0%	0.320	-
Paediatric patient	1	0.00 (-0.12 to 0.12)	-	1.000	-

MD, mean difference; CI, confidence interval; air-Q SP, air-Q self-pressurizing airway; LTS, laryngeal tube suction; Proseal, laryngeal mask airway Proseal.

Seven studies reported data comparing the ventilation score between the flexed and neutral neck positions.[4,10–12,14,16,17] The overall analysis revealed a significantly lower ventilation score in the flexed neck position (mean difference *−*0.74; 95% confidence interval *−*1.20 to *−*0.28; I^2^ = 98.8%; *P* = 0.002). Subgroup analyses according to the type of device revealed a significantly lower ventilation score with the i-gel (mean difference *−*1.25; 95% confidence interval *−*1.72 to *−*0.79; I^2^ = 91.5%; *P* < 0.001)[10,11,14] and the laryngeal tube suction (mean difference *−*1.50; 95% confidence interval *−*1.76 to *−*1.24; *P* < 0.001),[17] but no significant differences were found with the air-Q self-pressurizing airway[4] or the LMA Proseal.[11,16] Subgroup analysis by patient age revealed a significant decrease in the ventilation score during neck flexion in pediatric patients (mean difference *−*1.50; 95% confidence interval *−*1.63 to *−*1.37; I^2^ = 0.0%; *P* < 0.001), but not in adult patients.[10,12,17]

Five studies reported data comparing the ventilation score between the extended and neutral neck positions.[4,10,11,14,17] The overall analysis did not reveal any significant change in the ventilation score during neck extension (mean difference *−*0.11; 95% confidence interval *−*0.30 to 0.07; I^2^ = 92.3%; P = 0.221). Subgroup analysis revealed that neck extension only resulted in a significant decrease in the ventilation score with the air-Q self-pressurizing airway (mean difference *−*0.55; 95% confidence interval *−*0.69 to *−*0.41; *P* < 0.001).[4]

Four studies reported data comparing the ventilation score between the rotated and neutral neck positions.[4,11,14,17] The analysis of these studies revealed no significant change in the ventilation score between the two neck positions.

## Discussion

This meta-analysis shows that the flexed neck position significantly improves airway sealing but adversely affects ventilation and the fibreoptic view with most SADs. Although neck extension significantly reduced airway sealing, it did not affect ventilation or the fibreoptic view. Overall, neck rotation did not significantly affect SAD performance.

Changes in head and neck position can alter pharyngeal volume and shape, which can significantly affect the performance of SADs.[4,6,12] It might be expected that neck flexion would enhance the sealing function of SADs because it reduces the pharyngeal anteroposterior diameter by eliminating the longitudinal tension in the anterior pharyngeal muscles.[4,9,11,27] However, the reduction in space of the laryngeal inlet that occurs in the flexed neck position may provoke airway obstruction, resulting in poor ventilation and the need for higher airway pressures.[8,17] Conversely, neck extension increases the anteroposterior diameter of the pharynx by elevating the laryngeal inlet; thus, it may lead to decreased contact between the cuff and the mucosa, reducing the oropharyngeal leak pressure.[4,8] These changes in the pharyngeal structure according to head and neck position may affect the results of the overall analysis. However, substantial heterogeneity indicates that the results of the overall analysis may be more valid than the results of the subgroup analyses. In addition, the results from some of the subgroup analyses, according to the type of device and patient age, were inconsistent with the results of the overall analysis.

According to the subgroup analyses, the oropharyngeal leak pressure did not increase during neck flexion with the laryngeal tube suction. The study reported that the laryngeal tube suction has a large ellipsoid cuff and its anteroposterior diameter is larger than that of the LMA Proseal.[8] Due to these unique structural characteristics of the laryngeal tube suction, the reduced pharyngeal space that develops in the flexed neck position may not improve airway sealing around the cuff of this device. As evidence of this hypothesis, subgroup analysis revealed that the corbra perilaryngeal airway, which has a cuff of comparable size and shape to that of the laryngeal tube suction, also showed a smaller increase in the oropharyngeal leak pressure during neck flexion (mean difference 2.40 cmH_2_O), compared with the results of the overall analysis (mean difference 4.07 cmH_2_O).[8] Another result of the laryngeal tube suction in the flexed neck position was impaired ventilatory function. In fact, the greatest reduction in the ventilation score and the largest increase in the peak inspiratory pressure between the flexed and neutral neck positions was seen with the laryngeal tube suction. The previous study revealed that impaired ventilation in the flexed neck position was observed in 7 subjects with the laryngeal tube suction, and these subjects were excluded from the oropharyngeal leak pressure assessment.[8] Considering that there was no benefit in airway sealing and that ventilation became more difficult with the laryngeal tube suction during neck flexion, clinicians may need to exercise caution when choosing this device for procedures requiring the flexed neck position.

The ventilation score of the air-Q self-pressurizing airway did not significantly change in the flexed neck position compared with the neutral neck position. However, this does not necessarily indicate that partial airway obstruction does not occur, given the higher peak inspiratory pressure, and because it was more common to see the anterior epiglottis in the fibreoptic view with the air-Q self-pressurizing airway in the flexed neck position.[4] However, the partial airway obstruction that occurred with the air-Q self-pressurizing airway in the flexed position seems to be clinically insignificant.[4] As with the air-Q self-pressurizing airway, the i-gel also has a non-inflatable cuff, and a substantial decrease in the ventilation score was observed during neck flexion with this device.[10–12,14] In addition, the incidence of receiving the worst fibreoptic view rating was greatly increased as the vocal cords were not visible with the i-gel in the flexed neck position; however, this effect on the fibreoptic view was not observed with the air-Q self-pressurizing airway in the flexed neck position.[4,10–12,14,23] The difference in the fibreoptic view and ventilation score between the air-Q self-pressurizing airway and the i-gel in the flexed neck position may be attributable to the i-gel’s smaller area ventilating orifice, straighter airway tube, and thicker anteroposterior diameter compared with the air-Q self-pressurizing airway.[4] Although the air-Q self-pressurizing airway exhibited satisfactory performance during neck flexion, the i-gel exhibited worse performance during neck extension. Kim and colleagues demonstrated that the oropharyngeal leak pressure significantly decreased with the air-Q self-pressurizing airway in the extended neck position.[4] The sealing function of SADs, as assessed by the oropharyngeal leak pressure, is important for protecting the larynx and ensuring adequate ventilation.[28] Brimacombe and colleagues suggested that the oropharyngeal leak pressure should be greater than 10 cmH_2_O, as this is the approximate fluid pressure within the posterior pharyngeal space.[29] From the raw data provided by Kim and colleagues, an oropharyngeal leak pressure of less than 10 cmH_2_O was observed in 39% of cases with the air-Q self-pressurizing airway in the extended neck position.[4] In addition to inadequate airway protection, the air-Q self-pressurizing airway did not provide sufficient ventilatory function in the extended neck position, as evidenced by the decrease in the expiratory tidal volume and ventilation score.[4] Conversely, the i-gel provided clinically reasonable sealing and ventilatory function in the extended neck position relative to its function in the neutral neck position. This discrepancy between the function of these two devices may stem from the difference in cuff design. The cuff of the i-gel has a large anteroposterior diameter that can fill the wider pharyngeal space in the extended neck position, whereas the self-regulated cuff of the air-Q self-pressurizing airway seems to be insufficient to fill the increased pharyngeal space.[4] In light of these results, the i-gel may be preferable to the air-Q self-pressurizing airway during procedures requiring the extended neck position.

The LMA Proseal was the most commonly assessed device in the studies included in this meta-analysis.[8,11,15,16,24,25] The subgroup analysis revealed that the decrease in the oropharyngeal leak pressure with the LMA Proseal in the extended neck position was greater than the decrease in the oropharyngeal leak pressure seen in the overall analysis. As mentioned previously, the LMA Proseal has a smaller anteroposterior diameter and different cuff design than the laryngeal tube suction and corbra perilaryngeal airway.[8] The diameter and/or shape of the LMA Proseal may be associated with its reduced sealing function during neck extension. However, Park and colleagues reported that the LMA Proseal provided a reasonable mean oropharyngeal leak pressure value [18.5 (5.4) cmH_2_O] in the extended neck position and did not result in ventilation difficulty despite substantial oropharyngeal leak pressure reduction (mean difference *−*8.00 cmH_2_O).[8] In addition, no deterioration in the ventilation score or fibreoptic view with changes in head and neck position were observed with the LMA Proseal in our meta-analysis. Given the current evidence, the LMA Proseal could be utilised regardless of the head and neck posture.

Subgroup analyses by patient age showed significant differences in outcomes between pediatric and adult patients. In terms of the sealing function of SADs, the extent of the oropharyngeal leak pressure increase during neck flexion was comparable between both age groups, but the decrease in oropharyngeal leak pressure in pediatric patients during neck extension was less than the decrease in oropharyngeal leak pressure seen in adult patients. In the flexed neck position, the peak inspiratory pressure increased more than twice as much in pediatric patients as it did in adult patients, and the fibreoptic view and ventilation score greatly deteriorated in pediatric patients. This discrepancy according to patient age may be attributable to the anatomical differences in the upper airway between children and adults. Compared with adult patients, children have a larger tongue and epiglottis and more frequently have enlarged tonsils, which may result in a narrower pharyngeal space.[30] These anatomical features in pediatric patients seemed to worsen the negative effect of neck flexion on ventilation and alleviate the negative effect of neck extension on SAD sealing function. Therefore, neck extension may improve the fibreoptic view when using an SAD as a conduit for tracheal intubation in pediatric patients.

This meta-analysis has several limitations. Most importantly, the results indicate moderate to high levels of heterogeneity. This heterogeneity in the overall and subgroup analyses may be due to various factors, such as the type of device, patient age, study design, and use of neuromuscular blocking agents. To alleviate this limitation, we performed subgroup analysis according to the type of device and patient age. No subgroup analyse was performed separately by using of neuromuscular blocking agents because most of the studies that did not use muscular blocking agents were conducted in children. Second, the possibility of publication bias was observed in some of the overall and subgroup analyses. Thus, the outcomes of our meta-analysis may change with the addition of newly published articles and ongoing studies. Third, the number of articles included in the overall analysis was relatively small in terms of ventilation score data and subgroup analyses with the air-Q self-pressurizing airway and corbra perilaryngeal airway.[4,8] One study used ventilation quality scores,[1] and these scores were reversed for comparisons with the ventilation scores of other studies. Although the two methods are different, higher scores indicate better ventilation in both methods. Despite these weaknesses, to the best of our knowledge, the present study represents the first meta-analysis reporting changes in the performance of SADs in various head and neck positions.

This meta-analysis demonstrates that the reduced oropharyngeal leak pressure during neck extension does not result in a clinically significant impact on ventilation except with the air-Q self-pressurizing airway. Neck flexion negatively affects ventilation and the alignment between the SAD and glottis despite improving the sealing function of the SAD except with the air-Q self-pressurizing airway and LMA Proseal.

## Supporting information

S1 File Search terms. (DOCX)

S2 File Extracted data about the oropharyngeal leak pressure according to the flexed and neutral neck position. (CSV)

S3 File Extracted data about the oropharyngeal leak pressure according to the extended and neutral neck position. (CSV)

S4 File Extracted data about the oropharyngeal leak pressure according to the rotated and neutral neck position. (CSV)

S5 File PRISMA checklist. (DOC)
