# Supplementary material for: Influence of head and neck position on the performance of supraglottic airway devices: A systematic review and meta-analysis
Source: PLoS One. 2019 May 9;14(5):e0216673. doi: 10.1371/journal.pone.0216673 (PMC6508869; doi:10.1371/journal.pone.0216673)
Supplement: S1 File — (DOCX) [file pone.0216673.s001.docx]

<PubMed>

((((((((((head) OR neck)) AND ((positions) OR position))) OR extension) OR rotation) OR flexion)) AND (((((supraglottic airway) OR laryngeal mask) OR i-gel) OR air-Q) OR laryngeal tube))

(((((("head"[MeSH Terms] OR "head"[All Fields]) OR ("neck"[MeSH Terms] OR "neck"[All Fields])) AND (("Positions (Durh N C)"[Journal] OR "positions"[All Fields]) OR position[All Fields])) OR extension[All Fields]) OR ("rotation"[MeSH Terms] OR "rotation"[All Fields])) OR flexion[All Fields]) AND (((((supraglottic[All Fields] AND airway[All Fields]) OR ("laryngeal masks"[MeSH Terms] OR ("laryngeal"[All Fields] AND "masks"[All Fields]) OR "laryngeal masks"[All Fields] OR ("laryngeal"[All Fields] AND "mask"[All Fields]) OR "laryngeal mask"[All Fields])) OR i-gel[All Fields]) OR air-Q[All Fields]) OR (("larynx"[MeSH Terms] OR "larynx"[All Fields] OR "laryngeal"[All Fields]) AND tube[All Fields]))

<EMBASE>

(((head OR neck) AND (positions OR position)) OR extension OR rotation OR flexion) AND (supraglottic airway OR laryngeal mask OR i-gel OR air-Q OR laryngeal tube)

(('head'/exp OR head OR 'neck'/exp OR neck) AND (positions OR 'position'/exp OR position) OR 'extension'/exp OR extension OR 'rotation'/exp OR rotation OR 'flexion'/exp OR flexion) AND ((supraglottic AND ('airway'/exp OR airway) OR laryngeal) AND ('mask'/exp OR mask) OR 'i gel'/exp OR 'i gel' OR 'air q'/exp OR 'air q' OR laryngeal) AND ('tube'/exp OR tube) AND ([article]/lim OR [article in press]/lim)

<Cochrane>

Cochrane Central Register of Controlled Trials (CENTRAL)

'(((head OR neck) AND (positions OR position)) OR extension OR rotation OR flexion) AND (supraglottic airway OR laryngeal mask OR i-gel OR air-Q OR laryngeal tube)

<Web of Science>

(((((head OR neck) AND (positions OR position)) OR extension OR rotation OR flexion))) AND (((supraglottic airway OR laryngeal mask OR i-gel OR air-Q OR laryngeal tube)))
